# Relationship between retinal fluid characteristics and vision in neovascular age-related macular degeneration: HARBOR post hoc analysis

**DOI:** 10.1007/s00417-022-05716-4

**Published:** 2022-06-10

**Authors:** SriniVas Sadda, Nancy M. Holekamp, David Sarraf, Adel Ebraheem, Wenying Fan, Lauren Hill, Steve Blotner, Galin Spicer, Shamika Gune

**Affiliations:** 1 Doheny Eye Institute, 625 S Fair Oaks Ave, Suite 280, Pasadena, CA 91105 USA; 2 Pepose Vision Institute, Chesterfield, MO USA; 3 UCLA Stein Eye Institute, Ronald Reagan UCLA Medical Center, Los Angeles, CA USA; 4 Genentech, Inc., South San Francisco, CA USA

**Keywords:** Intraretinal, Fluid, Neovascular age-related macular degeneration, Retinal fluid, Subretinal, Vision

## Abstract

**Purpose:**

To evaluate the relationship between retinal fluid location, amount/severity, and vision with ranibizumab-treated neovascular age-related macular degeneration (nAMD).

**Methods:**

In the phase 3 HARBOR trial (NCT00891735), treatment-naive patients with nAMD received ranibizumab 0.5 or 2.0 mg through month 24. This post hoc analysis included eyes with subretinal fluid (SRF) and/or intraretinal fluid (IRF) at screening, baseline, or week 1, and optical coherence tomography data at months 12 and 24 (*n* = 917). Outcomes were best-corrected visual acuity (BCVA) change from baseline and proportion of eyes with 20/40 or better vision at months 12 and 24. Eyes were stratified by the location, amount, and/or severity of fluid.

**Results:**

At baseline, 86% and 63% of eyes had SRF and IRF, respectively. Among eyes with residual SRF, mean BCVA gains at each time point were greater in eyes with central versus noncentral SRF; location did not affect the odds of having 20/40 or better vision over 24 months. Eyes with 20/40 or better BCVA at month 12 had significantly lower SRF thickness versus eyes with worse vision; however, no difference was apparent at month 24. Vision was comparatively worse in eyes with residual IRF at months 12 and 24; location and severity did not appear to affect this outcome.

**Conclusion:**

Residual IRF was associated with worse vision outcomes, regardless of location/severity, whereas, despite continued treatment, residual SRF was not associated with worse vision outcome at 24 months, regardless of location/thickness. These data suggest complex relationships between residual fluid, severity, and vision.

**Supplementary Information:**

The online version contains supplementary material available at 10.1007/s00417-022-05716-4.


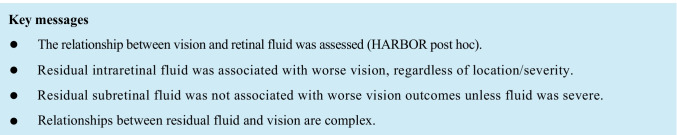


## Introduction

Antivascular endothelial growth factor (VEGF) therapy has dramatically reduced blindness caused by neovascular age-related macular degeneration (nAMD) [1, 2]. Based on the hypothesis that subretinal fluid (SRF) and/or intraretinal fluid (IRF) may be correlated with poor vision outcomes, a key goal of anti-VEGF therapy for nAMD has been complete resolution of retinal fluid to achieve a “dry” retina [3, 4]. Indeed, individualized regimens, such as treat-and-extend, adjust treatment intervals, with the aim of maintaining a dry retina or aggressively treating fluid when it returns [5–12].

Despite current treatment goals, recent evidence suggests that the relationships between SRF, IRF, and vision outcomes are complex and that complete fluid resolution may not necessarily lead to the best visual acuity outcomes [3, 4, 13–17]. For instance, a retrospective analysis of the VIEW study revealed that the presence of SRF at baseline was associated with improved best-corrected visual acuity (BCVA) from baseline over 1 year (5.3 letters gained with aflibercept), whereas the presence of IRF at baseline was not associated with improved BCVA [14]. Additionally, in a post hoc analysis of the phase 3 HARBOR trial of fixed monthly or pro re nata (PRN; or as-needed) ranibizumab for nAMD, improvements in BCVA over 24 months were greatest in eyes with residual SRF and resolved IRF, followed by eyes with resolved SRF and IRF. Eyes with residual SRF and IRF and eyes with residual IRF and resolved SRF had the worst outcomes [18].

Although previous studies have examined the effect of fluid presence and type (SRF or IRF) on visual responses to anti-VEGF therapy for nAMD [3, 13–15, 17], analyses of retinal fluid characteristics (location, thickness, severity) have been limited to 1 study. Post hoc analysis of data from the Comparison of Age-related Macular Degeneration Treatments Trials (CATT) at 1 [13], 2 [19], and 5 years [16] after initiation of anti-VEGF therapy in patients with nAMD evaluated the relationship between fluid location and vision outcomes in eyes with residual fluid. At years 1 and 2, BCVA was significantly better with versus without foveal SRF in multivariable models. At year 5, BCVA was significantly better with versus without foveal SRF in a univariate model but did not significantly differ in the multivariable model. Multivariable analyses at all time points showed that the presence of foveal IRF resulted in significantly worse BCVA versus extrafoveal IRF or no fluid. At years 1 [13] and 2 [19], BCVA was better among eyes with SRF thickness 1 to 25 µm compared with eyes that had no SRF or SRF thickness greater than 25 µm, whereas at year 5 [16], BCVA was similarly better in eyes with SRF thickness 1 to 25 µm and greater than 25 µm compared with eyes that had no SRF. CATT did not evaluate the relationship between vision and thickness of persistent SRF as a continuous measure, or the relationship between vision and severity of IRF. Such complex quantitative analyses may help further our understanding of the relationship between retinal fluid and vision in nAMD.

In this post hoc analysis of data from HARBOR, we evaluated not only the relationship between fluid location and vision, but also the relationship between both the thickness of persistent SRF (measured using a continuous scale) and the severity of IRF and vision outcomes after 24 months of ranibizumab therapy.

## Methods

### Design and setting

This was a post hoc analysis of the phase 3, randomized, multicenter, double-masked, active treatment–controlled HARBOR clinical trial (NCT00891735). The study design and primary outcomes have been described [11]. Briefly, patients aged 50 years or more with treatment-naive subfoveal nAMD were randomized to intravitreal ranibizumab 0.5 or 2.0 mg, administered monthly or PRN through month 24 [11]. After 3 monthly loading doses, patients randomized to the PRN arms were evaluated monthly and only received re-treatment if there was an at least 5-letter decrease in BCVA (assessed using standard Early Treatment Diabetic Retinopathy Study [ETDRS] protocols) from the previous visit or if there was evidence of disease activity on spectral-domain optical coherence tomography (SD-OCT) [11]. Disease activity was indicated by the presence of SRF, IRF, or subretinal pigment epithelium (RPE) fluid/detachment [20].

HARBOR was conducted in accordance with Good Clinical Practice, applicable US Food and Drug Administration regulations, and the Health Insurance Portability and Accountability Act. The study protocol was approved by institutional review boards and ethics committees; all patients provided written informed consent to participate and were compensated based on visit completion. Trial protocols are available online (https://vivli.org/).

### Post hoc analyses

The analyses included eyes with evidence of SRF and/or IRF at screening, baseline, or week 1 (hereafter referred to as “baseline”) and eyes with evaluable SD-OCT data at months 12 and 24 (*N* = 917; all treatment arms pooled). The presence of SRF and IRF was assessed using SD-OCT volumes (Cirrus OCT, 6 × 6 mm, foveal-centered, 512 × 128 A scan × horizontal B scans); evaluations were completed by 2 masked graders at the Doheny Image Reading and Research Lab, with an additional senior grader available to adjudicate any discrepancies. SRF was defined as evidence of a hyporeflective cavity occurring between the photoreceptor layer and the RPE. IRF was defined by hyporeflective cystoid intraretinal lesions in the inner nuclear layer and/or the outer nuclear layer, separated by thin reflective septa. For these analyses, sub-RPE fluid was not evaluated.

The proportion of eyes with baseline evidence of retinal fluid was evaluated by the location of IRF or SRF, defined as central or noncentral (superior, inferior, temporal, and/or nasal regions) using the modified ETDRS grid. SRF was characterized by mean thickness, evaluated at months 12 and 24. Using a previously validated method [21], IRF was categorized by severity as mild (cysts in ≤ 13 of the 128 B scans), moderate (cysts in 14–25 B scans), or severe (cysts in > 25 B scans) at baseline and months 12 and 24 (Supplementary Fig. 1). Outcomes of interest were the change from baseline in BCVA at months 12 and 24, stratified by IRF/SRF location and severity of IRF. Because SRF is a continuous variable, change from baseline in BCVA was not stratified by thickness. The proportion of eyes with 20/40 or better vision (BCVA ≥ 69 ETDRS letters) was evaluated by retinal fluid location, mean SRF thickness, and IRF severity at months 12 and 24. The odds of having 20/40 or better vision were calculated in these subgroups.

### Statistical analysis

Qualifying patients from all study arms were pooled to ensure a maximal available sample size for subgroup analyses. Observed data were used for all analyses, with no imputation for missing values. To adjust for differences in baseline BCVA across fluid location and severity subgroups, least square means were used to estimate changes in BCVA. Relationships between SRF location or IRF severity at months 12 and 24 and having 20/40 or better vision at each time point were evaluated using logistic regression, which included adjusting for baseline BCVA. Statistical analyses were performed using SAS software version 9.4 (SAS Institute, Inc., Cary, NC).

## Results

### Baseline vision by retinal fluid characteristics

Overall, 917 eyes receiving study treatment had evidence of SRF and/or IRF at baseline and had evaluable SD-OCT images at months 12 and 24. Among eyes with SRF (*n* = 785) and/or IRF (*n* = 577), most exhibited centrally located fluid (SRF, 61.2% [474/775]; IRF, 85.0% [487/573]). A higher proportion of eyes with IRF at baseline showed severe (55.4% [312/563]) versus moderate (23.1% [130/563]) or mild (21.5% [121/563]) IRF.

Of eyes with SRF at baseline, baseline BCVA was higher among eyes with central versus noncentral SRF (mean [95% confidence interval (CI)], 55.7 [54.6–56.8] letters vs. 53.0 [51.5–54.5] letters). Conversely, eyes with central IRF had lower baseline BCVA versus eyes with noncentral IRF (51.4 [50.3–52.5] letters vs. 57.8 [55.6–59.9] letters). Increasing IRF severity was associated with worse BCVA at baseline (mild, 57.8 [55.9–59.7] letters; moderate, 54.3 [52.3–56.2] letters; severe, 49.7 [48.2–51.1] letters; Supplementary Fig. 2).

Baseline characteristics by retinal fluid type are summarized in Supplementary Table 1.

### Relationship between SRF characteristics and vision outcomes

**Fig. 1:**
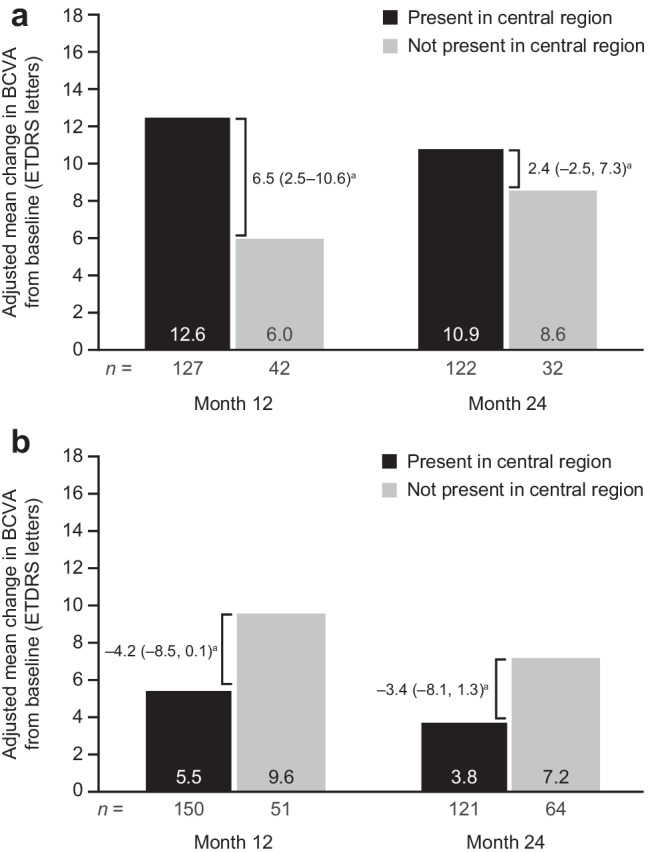
Mean change in best-corrected visual acuity (BCVA) by **a** subretinal fluid (SRF) location (central or noncentral) and **b** intraretinal fluid location (central or noncentral) at follow-up in eyes with residual SRF. Adjusted for baseline BCVA. *ETDRS*, Early Treatment Diabetic Retinopathy Study. ^a^Mean (95% confidence interval) difference (present minus not present)

Among eyes with residual SRF at months 12 or 24, BCVA gains from baseline at each time point were greater in eyes with central versus noncentral SRF (adjusted mean [95% CI] difference between central and noncentral ETDRS letters, 6.5 [2.5–10.6] vs. 2.4 [–2.5, 7.3]; Fig. 1).

**Fig. 2:**
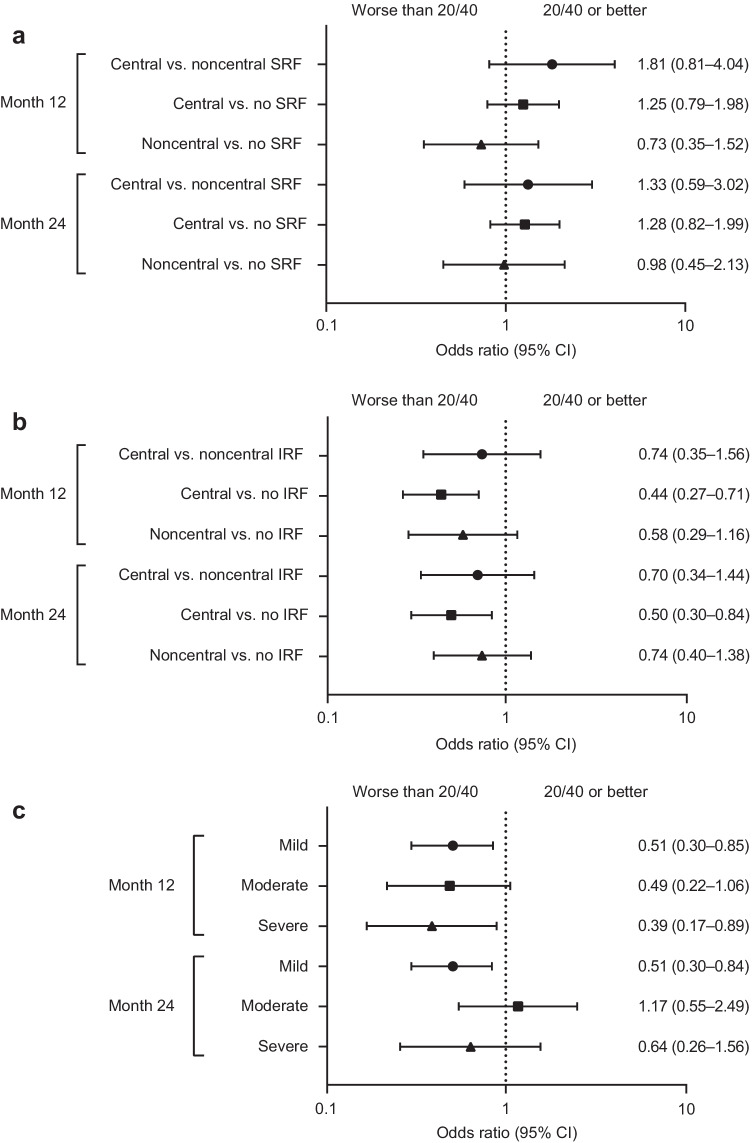
Odds of 20/40 or better vision by **a** subretinal fluid (SRF) location (central, noncentral, or no SRF), **b** intraretinal fluid (IRF) location (central, noncentral, or no IRF), and **c** IRF severity (mild, moderate, or severe). Analysis performed using logistic regression and adjusted for baseline best-corrected visual acuity. *CI*, confidence interval

**Fig. 3:**
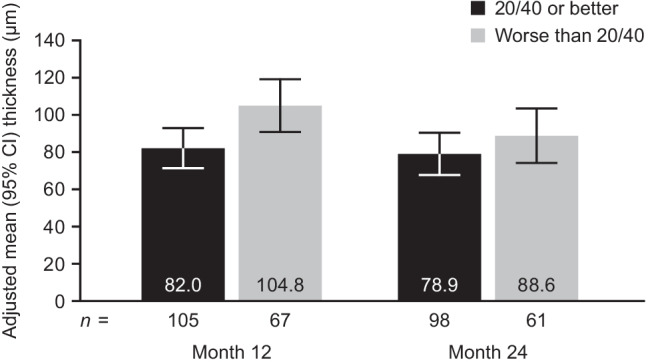
Relationship between 20/40 or better vision and subretinal fluid thickness at months 12 and 24. Analysis performed using logistic regression and adjusted for baseline best-corrected visual acuity. *CI*, confidence interval

In eyes with residual SRF, central versus noncentral SRF was not associated with having 20/40 or better vision at month 12 (odds ratio [OR] [95% CI], 1.81 [0.81–4.04]) or 24 (1.33 [0.59–3.02]; Fig. 2A). Similarly, central residual SRF versus no SRF (all SRF resolved) was not associated with having 20/40 or better vision at month 12 (1.25 [0.79–1.98]) or 24 (1.28 [0.82–1.99]; Fig. 2A). Among eyes with SRF present at month 12, average SRF thickness was significantly lower in eyes that had 20/40 or better vision versus eyes with worse vision (mean [95% CI], 82.0 μm [71.3–92.7] vs. 104.8 μm [90.7–118.9]; *p* = 0.016); however, eyes with residual SRF at month 24 had similar SRF thickness, regardless of vision (78.9 μm [67.6–90.2] vs. 88.6 μm [74.1–103.2]; *p* = 0.31: Fig. 3).

### Relationship between IRF characteristics and vision outcomes

**Fig. 4:**
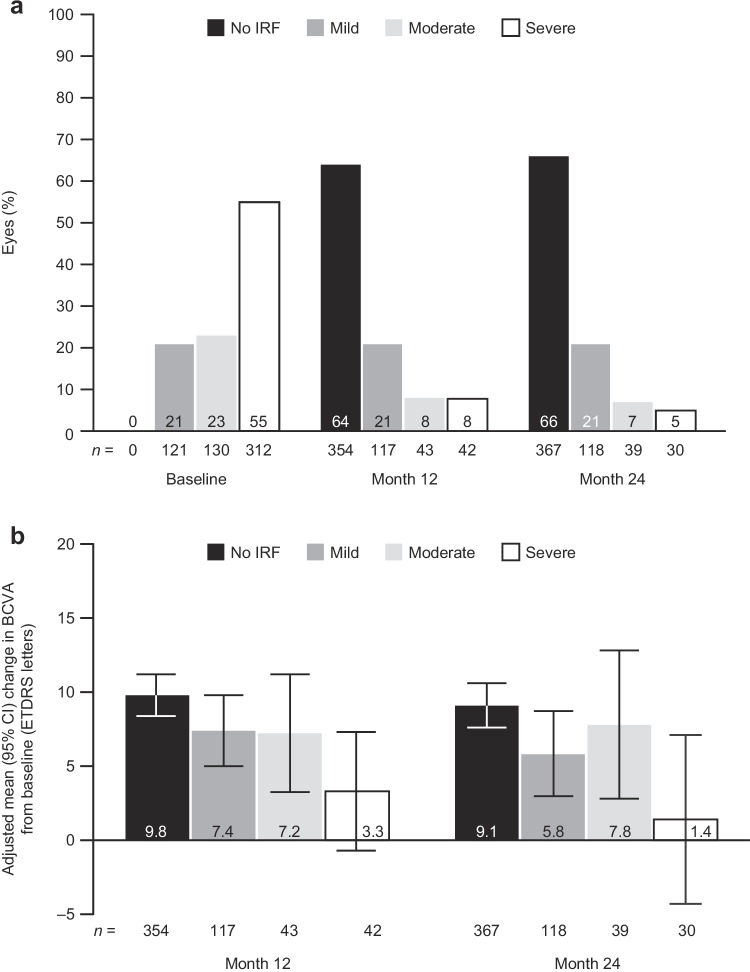
Changes in intraretinal fluid (IRF) severity with treatment and relationship to vision improvements. **a** Proportion of eyes with different IRF severity categories over time and **b** adjusted mean change in best-corrected visual acuity (BCVA; adjusted for baseline BCVA) by IRF presence and severity. *CI*, confidence interval; *ETDRS*, Early Treatment Diabetic Retinopathy Study

Among eyes evaluable for IRF severity, rates of severe IRF at baseline, month 12, and month 24 were 55.4% (312/563), 7.6% (42/556), and 5.4% (30/554), respectively (Fig. 4A). In these eyes that had IRF at baseline, the majority showed IRF resolution at months 12 (64%; 354/556) and 24 (66%; 367/554). In eyes with residual IRF, most eyes at months 12 and 24 had mild IRF (Fig. 4A). At month 12, we observed a trend for decreasing BCVA gains with increasing IRF severity; adjusted mean (95% CI) gain in BCVA from baseline decreased from 9.8 (8.4–11.2) ETDRS letters in eyes with no IRF at month 12 (354/562) to 7.4 (5.0–9.8) ETDRS letters in eyes with mild IRF (117/562) and 3.3 (–0.7, 7.3) ETDRS letters in eyes with severe IRF at month 12 (42/208: Fig. 4B). At month 24, adjusted mean (95% CI) gain in BCVA from baseline was 9.1 (7.6–10.6) ETDRS letters in eyes with no IRF at month 24 (367/566), 5.8 (3.0–8.7) ETDRS letters in eyes with mild IRF at month 24 (118/566), and 7.8 (2.8–12.8) and 1.4 (–4.3, 7.1) ETDRS letters in eyes with moderate (39/566) or severe (30/566) IRF at month 24, respectively, although there were small numbers of eyes in the groups with moderate or severe IRF.

Unlike SRF, the odds of having 20/40 or better vision were lower in eyes with central IRF versus eyes with no IRF at months 12 (OR [95% CI], 0.44 [0.27–0.71]) and 24 (0.50 [0.30–0.84]; Fig. 2B). Central versus noncentral IRF was not associated with having 20/40 or better vision at month 12 (OR [95% CI], 0.74 [0.35–1.56]) or 24 (0.70 [0.34–1.44]). Similarly, noncentral versus no IRF was not associated with 20/40 or better vision at month 12 (0.58 [0.29–1.16]) or 24 (0.74 [0.40–1.38]; Fig. 2B). The odds of having 20/40 or better vision at month 12 were lower in eyes with IRF versus no IRF. This was seen for all IRF severity levels at month 12 (OR [95% CI], vs. no IRF: mild, 0.51 [0.30–0.85]; moderate, 0.49 [0.22–1.06]; severe, 0.39 [0.17–0.89]; Fig. 2C). At month 24, the odds of having 20/40 or better vision were lower with mild IRF (0.51 [0.30–0.84]), whereas moderate and severe IRF were not associated with 20/40 or better vision (moderate, 1.17 [0.55–2.49]; severe, 0.64 [0.26–1.56]; Fig. 2C). The sample sizes for mild, moderate, and severe IRF at month 24 were 118, 39, and 30, respectively.

## Discussion

Our post hoc analyses of data from HARBOR are the first to evaluate more complex quantitative relationships between retinal fluid and vision at 12 and 24 months in eyes receiving ranibizumab for nAMD. Specifically, in addition to fluid location, we report novel findings concerning the relationships between vision outcomes and both the thickness of SRF (as a continuous measure) and the severity of IRF at these timepoints.

Overall, we found that in eyes that had residual SRF at months 12 and 24, those with centrally located residual SRF had significantly greater gains in BCVA at month 12 than eyes with noncentral SRF. At month 24, this trend was observed, but was not statistically significant. Conversely, eyes with residual central IRF had numerically lower, but not significantly different, BCVA scores versus noncentral IRF at both time points. The odds of having good vision (the equivalent of 20/40 Snellen vision) were similar for central SRF versus noncentral SRF and central SRF versus resolved SRF. In contrast, the odds of having good vision were lower for central IRF versus either noncentral IRF or resolved IRF. These data support observations made across multiple studies that residual SRF is not associated with worse BCVA outcomes at 2 years in eyes receiving consistent anti-VEGF treatment, whereas IRF is associated with poor vision [3, 4, 13–17].

Interestingly, we found that eyes with 20/40 or better vision had significantly lower SRF thickness compared with eyes that had worse than 20/40 vision at 12 months (the difference at 24 months was numerical only). This finding suggests that although mild (lower thickness) residual SRF may correlate with favorable visual outcomes, more severe (higher thickness) SRF may be associated with relatively worse outcomes, indicating that a more complex relationship exists between persistent SRF and visual acuity outcomes.

We also evaluated vision outcomes according to the presence of IRF and IRF severity. Residual IRF had a negative impact on vision, which increased with IRF severity. Specifically, eyes with no IRF had mean BCVA gains of 9 letters or more, whereas eyes with residual but mild or moderate IRF had mean BCVA gains of approximately 6 letters or more. Eyes with residual IRF graded as severe had the least gains in BCVA; however, these observations were based on a relatively small number of patients. Furthermore, eyes with mild IRF had greater odds of having vision worse than 20/40 at months 12 and 24 versus eyes with no IRF, as did eyes with central versus no IRF, regardless of severity.

Consistent with our study findings, a similar relationship between the location of residual SRF and vision outcomes was reported in CATT. After 5 years of anti-VEGF treatment, eyes with foveal SRF did not significantly differ in visual outcomes versus eyes without foveal SRF in multivariable models, but had better vision outcomes than eyes without foveal SRF in univariate models [16]. CATT also showed that IRF location at baseline was an important factor, with foveal IRF associated with worse BCVA outcomes after 1 and 2 years versus no IRF [13, 19]. Furthermore, after 5 years, residual extrafoveal IRF was still associated with worse visual outcomes [16]. In our study, we found no statistically significant effect of IRF location (central vs. noncentral) on the likelihood of 20/40 or better vision.

Clues to the mechanism underlying the association between residual SRF and more favorable vision outcomes may come from studies of retinal fluid in macular atrophy. The absence of SRF and the presence of intraretinal cysts at baseline were linked with the development of macular atrophy in HARBOR and CATT [22, 23]. The absence of SRF may be the result of atrophy due to complete loss of the RPE barrier, which may facilitate unimpeded passage of fluid through to the choroid. Therefore, the presence of SRF may indicate a viable and functioning, although impaired, RPE pump, whereas the absence of SRF may indicate complete loss of the RPE. Alternatively, SRF may be a sign of persistent type 1 macular neovascularization (MNV). A residual type 1 membrane might limit the development of atrophy by reducing areas of outer retinal ischemia, thereby supporting the RPE [22, 24]. Indeed, it has been suggested that MNV may arise as a compensatory mechanism in response to choriocapillaris loss observed during nAMD progression [25], although this needs confirmation in prospective studies. With regard to the negative impact of IRF on visual outcomes, the presence of IRF at baseline in nAMD may be a sign of preexisting retinal disruption and damage or more aggressive MNV, both of which could decrease the scope for improvements in visual acuity with treatment [17].

The vision outcomes observed in HARBOR were achieved when eyes with evidence of SRF received ongoing treatment, either on a monthly basis or per prespecified disease activity criteria [22]. Our findings do not suggest that exudation should be left untreated; rather, they suggest that as long as patients are receiving regular treatment and demonstrating maintenance of meaningful vision outcomes, residual SRF may not preclude good visual outcomes, at least through 2 years. As long as vision outcomes are acceptable with a given treatment regimen, residual SRF in the absence of IRF may indicate stable disease and may be tolerable. Conversely, residual IRF may be of concern regarding visual outcomes because it may be a marker of degenerative damage to the retina and an indicator of poorer visual potential.

A number of study limitations may impact the applicability of our findings. This was a post hoc analysis of pooled data from eyes treated with monthly or PRN ranibizumab; our analyses were not adjusted to account for differences in the number of injections received across treatment arms. It should be noted that all 4 treatment groups in HARBOR achieved clinically meaningful improvements in BCVA over 12 and 24 months [11, 20]. Most of the analyses in this study were not corrected for the presence of IRF in eyes with SRF, and vice versa. Moreover, despite adjusting for baseline BCVA, eyes with residual SRF at months 12 and 24 had higher baseline BCVA than eyes with resolved SRF, suggesting some imbalance that could impact the comparability of these groups. Another limitation is that eyes with subfoveal fibrosis and/or atrophy were not excluded from the analysis; however, it could be argued that the inclusion of these eyes was reflective of what might be encountered in real-world clinical practice. Finally, our analysis could not determine the impact of the duration or persistence of edema on visual acuity; further study is needed to evaluate whether there is any impact.

This post hoc analysis of HARBOR found that in eyes with residual fluid, vision gains after 12 and 24 months of ranibizumab treatment were more favorable among eyes with residual SRF located in the central macular region. Conversely, IRF located in the central region was associated with poor vision outcomes, and a trend for decreasing BCVA gains with increasing IRF severity was also noted. Although we found a strong relationship between the type and location of retinal fluid and visual gains achieved with ranibizumab treatment, the observations regarding 20/40 or better vision were more nuanced. Specifically, we found that the odds of having 20/40 or better vision were similar, regardless of SRF presence and location. In contrast, IRF presence in the center versus no IRF was significantly associated with greater odds of having vision worse than 20/40. It is important to note that patients in HARBOR were treated for the entire study duration of 24 months with either a monthly or PRN regimen, wherein fluid presence on OCT was a re-treatment criterion. Therefore, our findings do not suggest that exudation in nAMD should be left untreated. Rather, these data demonstrate a complex relationship between retinal fluid and vision outcomes, suggesting that “dry” retinas may not necessarily correlate with superior vision gains through at least 2 years. Moreover, although we continue to advocate the treatment of SRF in nAMD, the presence of SRF may indicate a greater viability of retinal pathoanatomy and a greater potential for vision improvement with continued therapy. To improve nAMD treatment paradigms and further reduce rates of vision loss, a more sophisticated understanding of the pathophysiological mechanisms underlying exudation in nAMD is required.

## Supplementary Information

Supplementary file1 (PDF 181 KB)

Supplementary file2 (PDF 189 KB)

Supplementary file3 (PDF 215 KB)

## Data Availability

For eligible studies, qualified researchers may request access to individual patient level clinical data through a data request platform. At the time of writing, this request platform is Vivli. https://vivli.org/ourmember/roche/. For up-to-date details on Roche’s Global Policy on the Sharing of Clinical Information and how to request access to related clinical study documents, see here: https://go.roche.com/data_sharing. Anonymized records for individual patients across more than one data source external to Roche cannot, and should not, be linked due to a potential increase in risk of patient re-identification.
