# Supplementary material for: Relationship between retinal fluid characteristics and vision in neovascular age-related macular degeneration: HARBOR post hoc analysis
Source: Graefes Arch Clin Exp Ophthalmol. 2022 Jun 10;260(12):3781–9. doi: 10.1007/s00417-022-05716-4 (PMC9666309; doi:10.1007/s00417-022-05716-4)
Supplement: Supplementary file 1 — Supplementary file1 (PDF 181 KB) [file 417_2022_5716_MOESM1_ESM.pdf]

Supplementary Table 1 Baseline demographics and ocular characteristics by retinal fluid type

Variable	SRF (n = 785)	IRF (n = 577)
Mean age, years (SD)	77.5 (8.2)	79.3 (7.9)
Male, n (%)	336 (42.8)	223 (38.6)
Race, n (%)		
American Indian or Alaska Native	2 (0.3)	0 (0)
Asian	11 (1.4)	8 (1.4)
Black or African American	3 (0.4)	2 (0.3)
Native Hawaiian or other Pacific Islander	2 (0.3)	2 (0.3)
White	760 (97.6)	561 (97.9)
Hispanic or Latino, n (%)	23 (2.9)	16 (2.8)
Mean BCVA, ETDRS letters (SD)	54.6 (12.5)	52.4 (12.5)
Mean CST, μm (SD)	382.6 (120.4)	406.6 (129.8)
Mean CFT thickness, μm (95% CI)	347.2 (143.1)	381.1 (148.8)
Atrophy, n (%)	92 (12.4)	90 (16.9)
Central SRF, n (%)	474 (61.2)	NA
Mean SRF maximal thickness, μm (SD)	158.5 (103.1)	NA
Mean SRF volume, μm^3 (SD)	2.0 (5.5)	NA
Central cyst, n (%)	NA	487 (85.0)
Cyst severity, n (%)		
Mild	NA	121 (21.5)
Moderate	NA	130 (23.1)
Severe	NA	312 (55.4)

BCVA, best-corrected visual acuity; *CI*, confidence interval; *CST*, central subfield thickness; *CFT*, central foveal thickness*; ETDRS*, Early Treatment Diabetic Retinopathy Study; *IRF*, intraretinal fluid; *SD*, standard deviation; *SRF*, subretinal fluid.
