# Supplementary material for: Relationship between retinal fluid characteristics and vision in neovascular age-related macular degeneration: HARBOR post hoc analysis
Source: Graefes Arch Clin Exp Ophthalmol. 2022 Jun 10;260(12):3781–9. doi: 10.1007/s00417-022-05716-4 (PMC9666309; doi:10.1007/s00417-022-05716-4)
Supplement: Supplementary file 2 — Supplementary file2 (PDF 189 KB) [file 417_2022_5716_MOESM2_ESM.pdf]

Supplementary Fig. 1 Assessing subretinal and intraretinal fluid (IRF) location and IRF severity. (**a**) Modified Early Treatment Diabetic Retinopathy Study for assessing fluid location. (**b**) Grading IRF severity ("cysts").

a

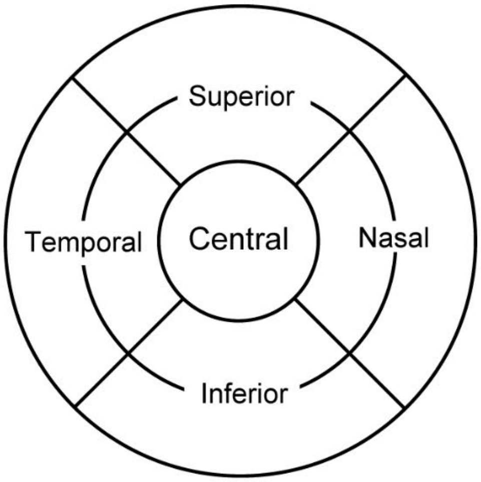

b

Mild	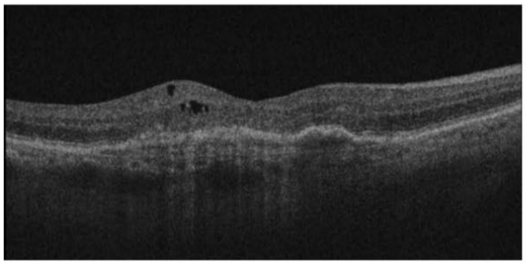	Cysts in ≤ 13 B scans
Moderate	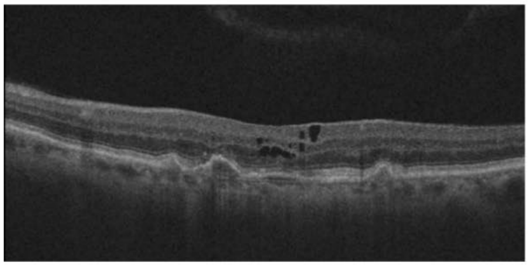	Cysts in 14–25 B scans
Severe	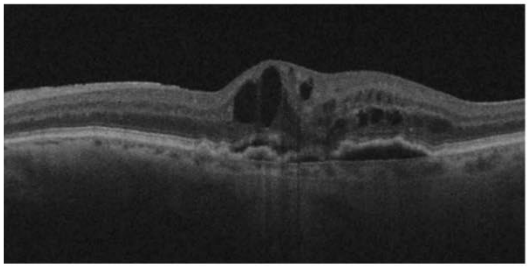	Cysts in > 25 B scans
