# Supplementary material for: Relationship between retinal fluid characteristics and vision in neovascular age-related macular degeneration: HARBOR post hoc analysis
Source: Graefes Arch Clin Exp Ophthalmol. 2022 Jun 10;260(12):3781–9. doi: 10.1007/s00417-022-05716-4 (PMC9666309; doi:10.1007/s00417-022-05716-4)

Supplementary Fig. 2 Relationship between baseline best-corrected visual acuity (BCVA) and (**a**) baseline fluid location and (**b**) baseline intraretinal fluid (IRF) severity. *CI*, confidence interval; *ETDRS*, Early Treatment Diabetic Retinopathy Study; *SRF*, subretinal fluid.

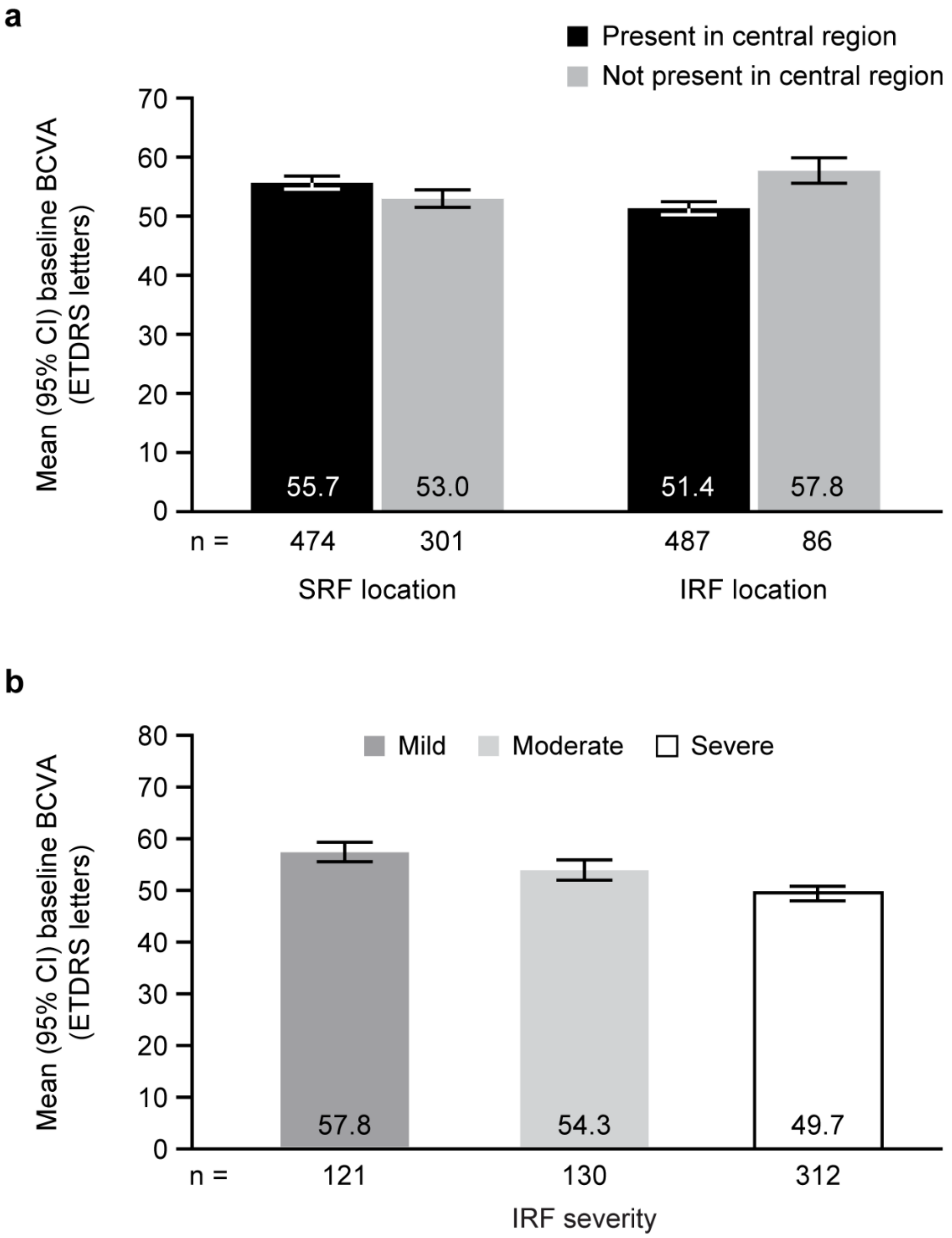

Supplement: Supplementary file 3 — Supplementary file3 (PDF 215 KB) [file 417_2022_5716_MOESM3_ESM.pdf]
